# The intellectual base and research fronts of LGR5: a bibliometric analysis

**DOI:** 10.3389/frma.2025.1644408

**Published:** 2025-10-21

**Authors:** Rong Ding, Zemin Tang, Rong Xu, Zhiming Deng

**Affiliations:** ^1^Department of Pathology, Changde Hospital, Xiangya School of Medicine, Central South University (The First People's Hospital of Changde City), Changde, Hunan, China; ^2^Department of Endocrinology, Changde Hospital, Xiangya School of Medicine, Central South University (The First People's Hospital of Changde City), Changde, Hunan, China

**Keywords:** LGR5, cancer stem cells, bibliometric, CiteSpace, VOSviewer, tumor microenvironment

## Abstract

**Background:**

Leucine-rich repeat-containing G-protein-coupled receptor 5 (LGR5) is involved in canonical Wnt/β-catenin signaling and is a marker of stem cells in several tissues. It plays an important role in self-renewal, metastasis, and chemoresistance of tumors. The plasticity and potential of LGR5 (+) cancer stem cells could provide therapeutic targets for cancer. However, the data in this field is very limited and requires further investigation.

**Methods:**

This study aimed to explore the status and evolutionary trends of LGR5 research using bibliometric analysis. In total, 2,187 publications were retrieved from the Web of Science Core Collection. The period covered by the articles was from 1999 to 2023. CiteSpace, VOSviewer, R software, and Bibliometric Online Analysis Platform were used for bibliometric analysis and visualization.

**Results:**

The USA was the most productive country, with the highest centrality and largest single-country publications. The Netherlands was the earliest country to conduct LGR5 research. Cleavers, H from the Hubrecht Institute (KNAW) of the Netherlands was the most influential author as measured by H, G, and M-index values and contributions to the number of publications and citations. Intestinal stem cells were a hot topic, while keywords “LGR5 (+) stem cells,” “inflammation,” and “tumor microenvironment” exhibited the strongest burst in recent years, indicating a significant research focus in the future. Additionally, targeting LGR5 (+) stem cells in a specific phase of cancer and in combination with tumor microenvironment (TME) combination could be a future hotspot.

**Conclusion:**

Research on LGR5 continues to develop through active global efforts. This study offers a comprehensive analysis of LGR5 using bibliometric and visual methods, highlighting current research hotspots and potential directions for researchers interested in the field.

## Introduction

G-protein-coupled receptors (GPCRs) are membrane proteins that transmit extracellular signals into the cell, typically in response to hormones and neurotransmitters. Disfunctions in GPCRs have been implicated in a wide range of diseases, making them key targets for both academic research and drug development (Kumar et al., 2014). Leucine-rich repeat-containing G-protein-coupled receptor 5 (LGR5) is a well-known Wnt target gene, is pivotal for the regulation of stem cells and maintenance of tissue homeostasis. In adult intestinal crypts and colon cancer (de Lau et al., 2014) LGR5 marks cycling crypt base columnar cells, which are considered as true intestinal stem cells capable of differentiating into all types of intestinal epithelial cells. In the bulge of telogen hair follicles, LGR5 expression is confined to actively cycling cells, suggesting LGR5 serves as a global marker of adult stem cells (Hata et al., 2018; Haegebarth and Clevers, 2009). Importantly, LGR5 has a dual function: on the one hand, it serves as a marker of proliferative stem cells; on the other hand, it functions as a receptor for R-spondins (Rspo), thereby potentiating Wnt/β-catenin signaling and further amplifying stem cell maintenance and tumorigenic potential (Wang et al., 2025).

Additionally, LGR5 is a significant prognostic factor for colorectal cancer (CRC) (Chen et al., 2014). In hepatocellular carcinoma, LGR5 overexpression identifies a distinct subgroup that may inform precise staging and treatment decisions (Effendi et al., 2014). Increasing evidence suggests that endogenous LGR5-positive cell population contributes to tumor initiation, progression, and metastasis. Therefore, targeted drug delivery systems or genome editing strategies directed at LGR5-positive cancer cells may provide novel anticancer approaches (Xu et al., 2019). However, the underlying molecular mechanisms of LGR5-mediated tumor promotion or suppression are not fully understood, and further research is necessary to elucidate its role in maintaining homeostasis and tumor growth.

The number of studies related to LGR5 has been steadily increasing over the years. Compared with traditional approaches, bibliometric analysis provides an efficient means of summarizing and examining large volumes of literature and complex data, thereby generating insights in a concise manner. It also serves as a valuable resource for researchers seeking to initiate or extend studies in this field. As a statistical and mathematical approach, bibliometric analysis is particularly useful for identifying research hotspots and emerging trends. Tools such as CiteSpace and VOSviewer have been widely applied to map knowledge domains and to visualize co-occurrence patterns and co-authorship networks. To date, no comprehensive bibliometric analysis of LGR5 has been published. Therefore, we performed a bibliometric analysis to objectively characterize the general landscape, knowledge base, hotspots, and emerging trends in LGR5 research. Our objectives were to: (1) quantify and identify the general information on LGR5 research by investigating annual publications and citations, as well as the contributions from countries, institutions, journals, and authors; (2) analyze the most citated references to structure the knowledge base in LGR5 research; (3) perform an analysis of keywords and co-citation references to identify the hotspots and potential directions in LGR5 research. We believe that a deeper understanding of the current knowledge structure, emerging cutting-edge fields, and popular topics in LGR5 research will provide valuable guidance for future studies.

## Materials and methods

### Data screen and selection criteria

The Web of Science Core Collection (WOSCC) is a highly influential multidisciplinary academic literature abstract index database, which originated in 1985. It encompasses Science Citation Index Expanded, Social Sciences Citation Index, and Arts and Humanities Citation Index. Two of its major strengths are reference tracing and citation reporting, both of which are essential for identifying research outputs and trends within a given field (Huang et al., 2021). In addition to enabling access influential academic journals, books, and citation networks, WoSCC employs a strict selection process grounded in Bradford's low of bibliometrics. This ensures the inclusion of only core academic journals and major international conferences across all disciplinary fields (Ou et al., 2023).

**Figure 1:**
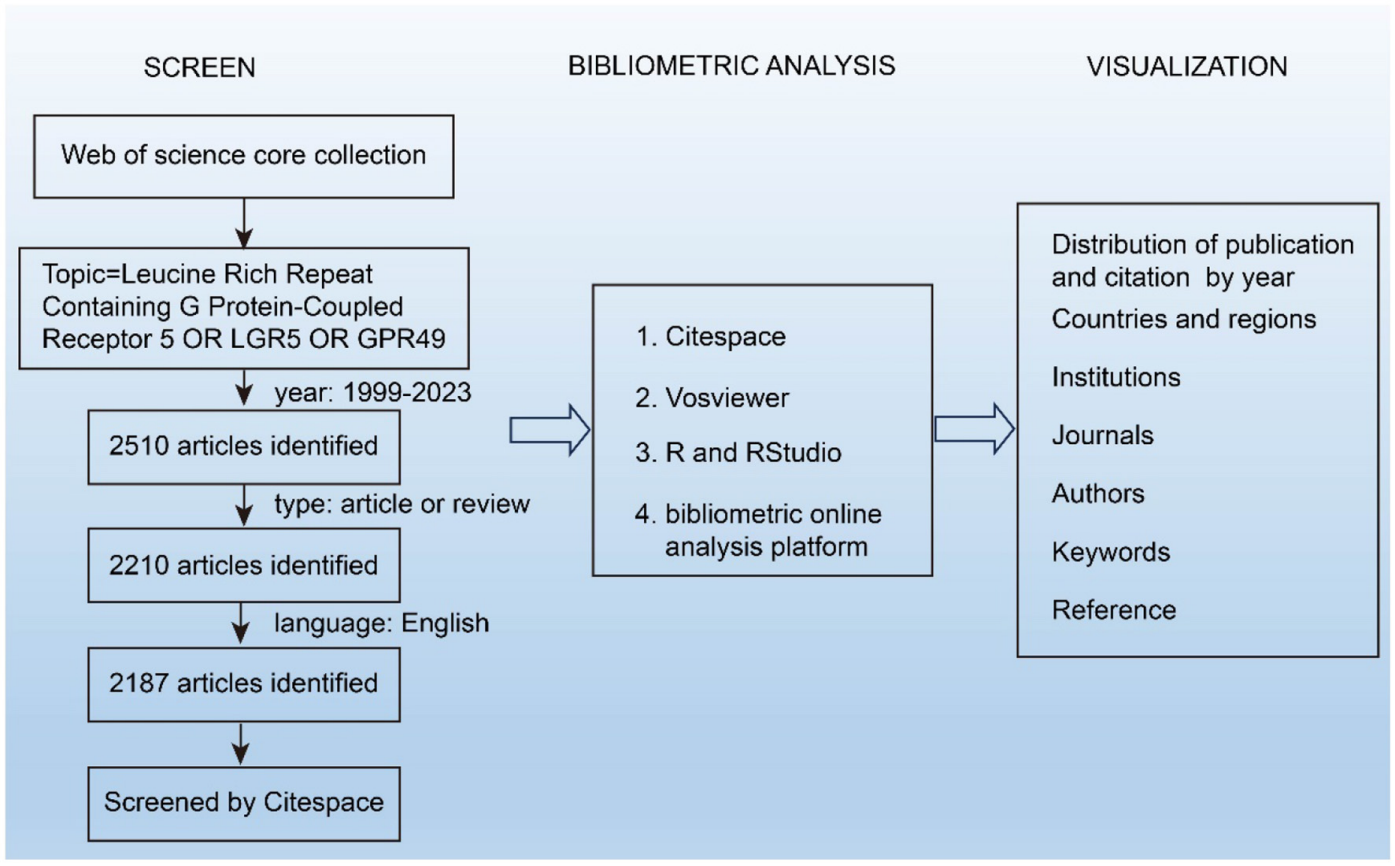
Detailed flowchart steps of the search strategy in the screening of publications.

In this study, the search term “Leucine Rich Repeat Containing G Protein-Coupled Receptor 5 OR LGR5 OR GPR49” was used to retrieve relevant literature. The time span was set from 1999 to 2023, and the article types were refined to articles and reviews. The language used was confined to English. A total of 2,187 articles were obtained from the WOSCC on September 02, 2024. Due to the time lag between data collection and the submission/review process, the analysis was restricted to publications from 2000 to 2024. This restriction was applied to ensure consistency and reproducibility of the results. The detailed search strategy is shown in Figure 1.

### Data collection and analysis

Raw data were downloaded from the WOSCC database in text or tab-delimited file formats. Duplicate records were removed using CiteSpace, and the cleaned data were merged into a text file (download_xx) for use in CiteSpace (version 6.2. R4), VOSviewer (version 1.6.17), an online bibliometric analysis platform, and R software (version 4.1.3) for systematic analysis.

Annual publications and citations were retrieved from citation reports in the WoSCC database. CiteSpace developed by Dr. Chaomei Chen, is a scientific literature analysis tool that identifies research structures and development trends within a given field. It assesses co-occurrence and centrality in cooperation networks to identify key nodes: nodes size represents the number of documents while the color and proportion of the outermost ring indicate centrality, thereby highlighting node importance. Citation burst of terms reflects active or emerging research fronts; the longer the red segment, the longer the keyword's popularity and the stronger its frontier status. The VOSviewer software was used to visualize scientific landscapes by network/time-dependent overlay/density using linlog/modularity methods. Weights were determined by citation frequency or publication counts, and the colors represented publication years. The terms were further clustered according to the selected analytical mode. The RStudio platform was used to run the Bibliometrix and Biblioshiny packages, which provide comprehensive bibliometric research tool for mining knowledge structures and social networks (Ogunsakin et al., 2022). The Bibliometrics Online Analysis Platform was additionally applied to examine collaboration networks between countries or regions. Microsoft Excel was used for basic data management, including importing, classification, and table generation.

### Bibliometric indicators

To evaluate the contributions of authors in the LGR5 research field, we calculated the H, G, and M indices. The H-index measures both productivity and citation impact, reflecting the cumulative significance of a researcher's output. The G-index was developed to address the limitation and inaccuracy of the H-index in small-sample publications, and considers the performance of the cited articles while retaining all the advantages of the H-index, particularly in small publication samples, by considering the performance of highly cited articles while retaining the advantages of H-index, thus providing a more comprehensive assessment of research achievement (Xiao et al., 2022). M-index is calculated by dividing the H-index by the number of years since the first article in the dataset, Allowing for comparisons of research productivity over time and accounting for early-career contributions (Pang et al., 2022; Musbahi et al., 2022).

### Journal impact evaluation

Journal influence was assessed using the Impact Factor (IF), as reported by *Journal Citation reports* (JCR), published by Clarivate Analytics (Huang et al., 2021). JCR classifies journals into different thematic categories and ranks them into quartiles (Q1–Q4) based on IF values.

### Research ethics

There are no animal experiments or human tissues in our study, and the data used in this study were retrieved from publicly available sources.

### Statistical analysis

CiteSpace was employed to analyze institutions, journals, reference timelines, citation bursts (by country, references, and keywords), and keyword networks. In these visualizations, node size corresponds to citation frequency, while links represent co-occurrence relationships. Citation corresponds to citation frequency, while links represent co-occurrence relationships. Citation bursts highlight the significance of specific terms in particular studies. VOSviewer was used to generate overlay and time-dependent visualization maps. Bibliometrics Online Analysis Platform was used for analysis of collaboration between countries or regions. R Bibliometrix was utilized to examine the distribution of publications and the authors contributions. GraphPad 9.5 was used for descriptive statistical analyze of institutional data and for generating diagrams.

## Results

### Annual publication and citation

**Figure 2:**
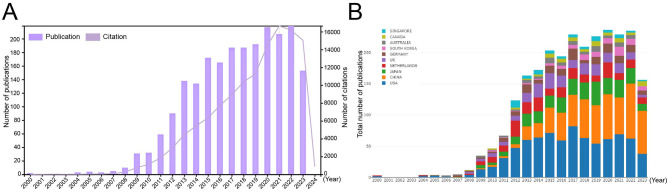
Annual publication number and citation of LGR5 related research **(A)**. Composition ratio of the top 10 countries by publication number **(B)**.

A total of 2,187 publications on LGR5, published between 1999 and 2023, were retrieved from the WoSCC database, including 2014 articles (92.1%) and 197 reviews (7.9%). The distribution of publication numbers by year showed a steadily increasing trend (Figure 2A). Between 2000 and 2009, only 29 publications were recorded; however, the number of publications increased rapidly between 2009 and 2015, and then remained relatively stable after 2015. In contrast, the number of citations continued to rise steadily each year. An analysis of the top ten contributing countries revealed that the USA led in publication output until 2017. From 2018 to 2021, the numbers of publications from the USA and China were comparable. Since 2022, China had shown remarkable growth, surpassing the USA to become the country with the highest number of publications (Figure 2B).

### Country and institution analysis

**Figure 3:**
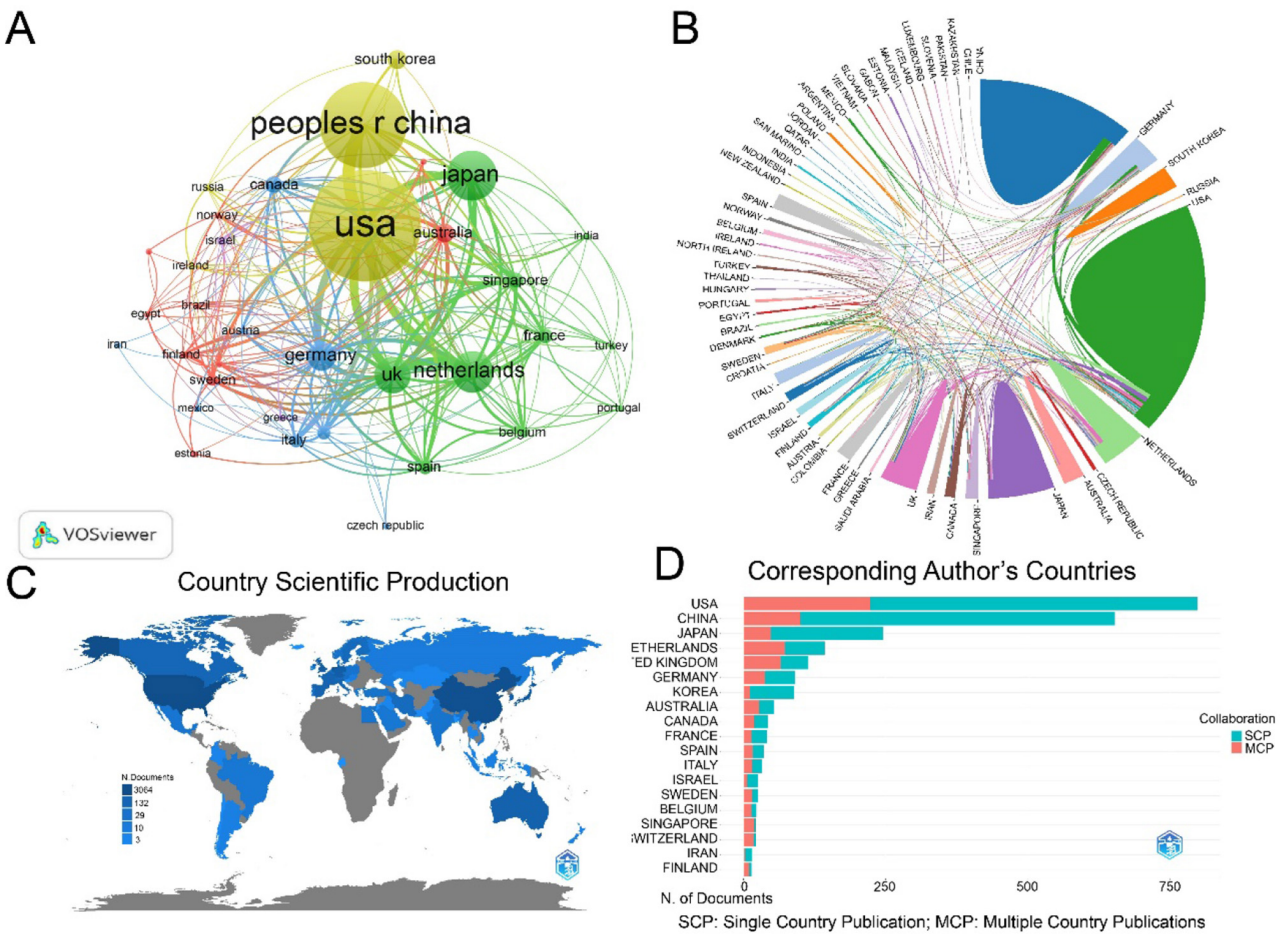
Country/regions analysis. **(A)** The co-occurrence map or countries in LGR5 research. Node size reflects frequency, colors denote thematic clusters, and line thickness indicates co-occurrence strength. **(B)** Collaboration networks between countries/regions. **(C)** Geographical distribution of LGR5 research. **(D)** The ratio of SCP and MCP of corresponding author's countries.

**Figure 4:**
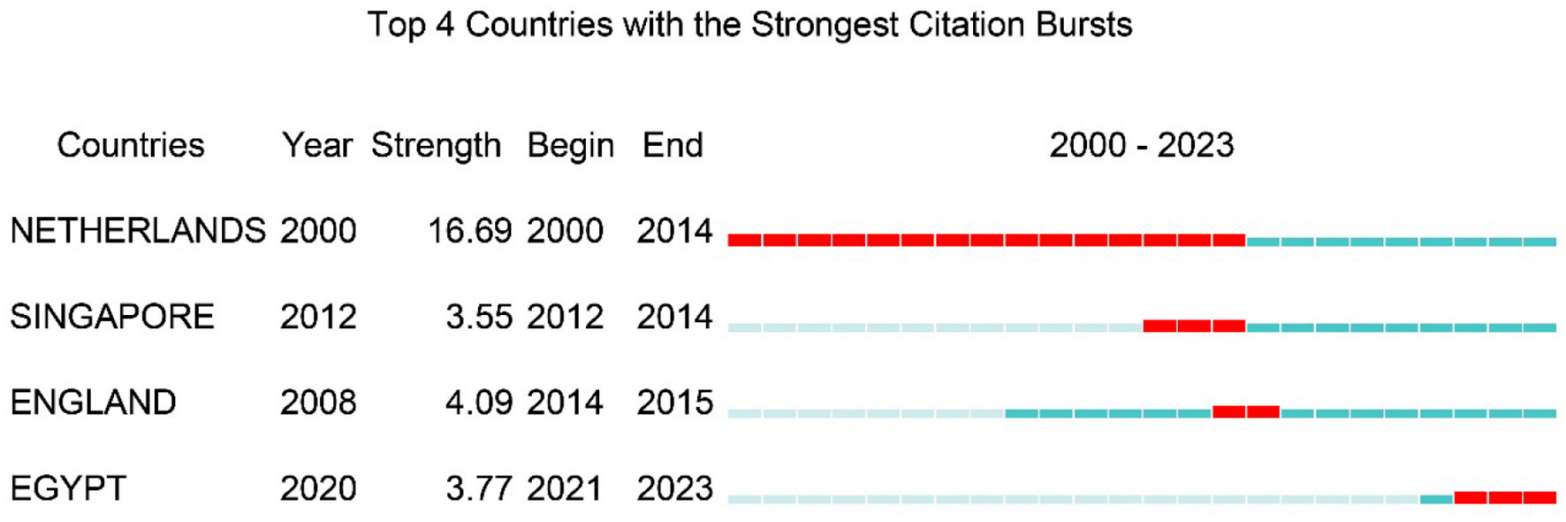
The strongest citation bursts of countries. The red bars present citation burstness and the blue bars means the reference had been published.

The 2,187 publications involved co-authors from 400 countries/regions and 8,213 institutions. The USA produced the largest number of articles, followed by China, Japan, the Netherlands, the United Kingdom, Germany, Korea, Australia, Canada, and France (Figure 3A). The international collaboration visualization map provided an intuitive display of global scientific cooperation, showing the closest partnership between China and the USA, followed by Japan, the Netherlands, and the UK (Figure 3B). Geographically, most LGR5-related research articles originated from the USA and China (Figure 3C). Among the top ten most productive countries, the USA had the highest centrality and citations counts. Moreover, the USA published the most articles without any collaborations with other countries, whereas the Netherlands and the UK tended to collaborate with other countries to publish articles (Figure 3D, Table 1). These results indicated the leading position of the USA in LGR5-related research. Furthermore, the Netherlands had the strongest citation bursts, while Egypt was the latest country to conduct LGR5 related research (Figure 4).

**Table 1:** The most productive 10 countries with documents on LGR5 research.

**No**.	**Countries**	**Documents**	**Citations**	**Centrality**	**SCP**	**MCP**	**MCP_Ratio**
1	USA	831	58,746	0.38	599	232	0.279
2	China	556	11,918	0.04	470	86	0.153
3	Japan	288	10,792	0.14	232	56	0.194
4	Netherlands	228	41,082	0.19	112	116	0.509
5	United Kingdom	149	14,015	0.21	64	85	0.571
6	Germany	141	9,266	0.19	82	59	0.417
7	Korea	80	1,511	0.02	70	10	0.127
8	Australia	79	3,238	0.13	39	40	0.512
9	Canada	68	3,451	0.04	38	30	0.441
10	France	62	4,244	0.02	41	21	0.333

**Figure 5:**
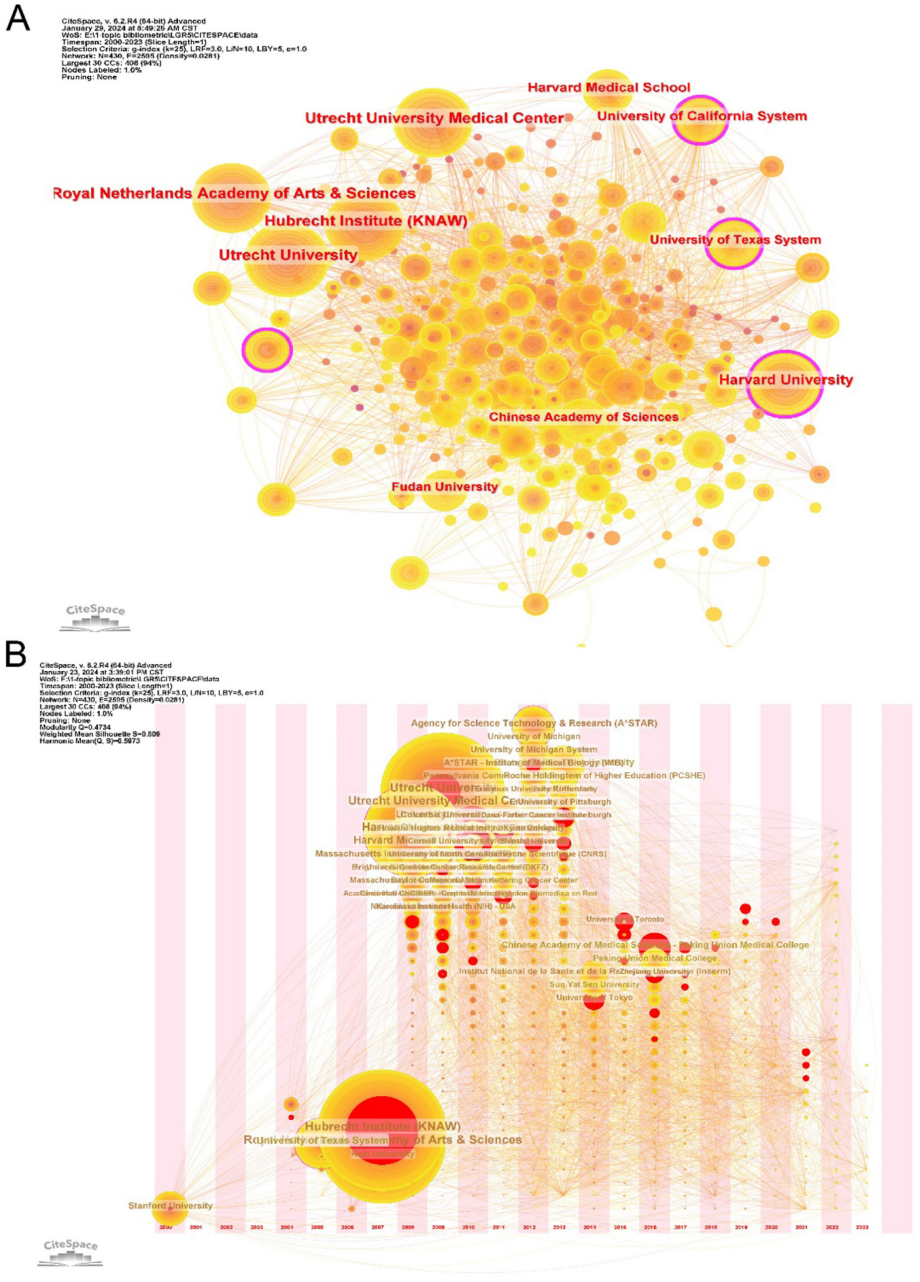
The co-occurrence map **(A)** and time zone map **(B)** of institutions in LGR5 research. The size of notes present the co-occurrence frequencies and the outer ring of nodes colored with purple means its hight betweenness centrality (>0.1).

The top ten institutions by publication output are listed in Table 2. The Hubrecht Institute (KNAW) in the Netherlands ranked first with 140 articles. Although the Antoni Van Leeuwenhoek Hospital did not among the top ten most productive institutes, it achieved more than 10,000 citations, indicating significant academic influence. The co-occurrence network of institutions showed the top ten productive institutions. The nodes of Harvard University, the University of California System, and the University of Texas System colored purple round presented significant centrality (≥0.10), which is usually regarded as a valuable node in a network (Figure 5A). Over time, emerging institutions became increasingly involved in LGR5 research (Figure 5B). The time-zone map further showed that institutions such as the Hubrecht Institute (KNAW), Utrecht University Medical Center, and the Royal Netherlands Academy of Arts & Sciences published the largest number of articles in 2006 and 2009. Since 2009, Chinese institutions such as the Peking Union Medical College, Zhejiang University, and the University of Chinese Academy of Sciences have emerged as leading contributors in this field.

**Table 2:** Top 5 institutions with the largest number of documents.

**No**.	**Institutions**	**Documents**	**Citations**	**Total link strength**	**Country**
1	Hubrecht Institute (KNAW)	140	12,919	6,226	Netherlands
2	Royal Netherlands Academy of Arts and Sciences	138	1,072	1,618	Netherlands
3	Utrecht University	135	3,626	2,621	Netherlands
4	Utrecht University Medical Center	128	32,109	13,368	Netherlands
5	Harvard University^a^	106	4,605	2,689	USA
6	Harvard Medical School	68			USA
7	University of California System^a^	65	2,994	1,922	USA
8	University of Texas System^a^	58	2,204	1,483	USA
9	Chinese Academy of Sciences	56	1,422	1,403	China
10	Fudan University	51	1,072	1,618	China
The institutions with total citation over 10,000					
:	:	:	:	:	:
119	Antoni Van Leeuwenhoek Hosp	9	11,490	5,322	UK

^a^It means that the centrility value was greater than 0.1, indicating its imprtant contribution in LRG5 research field.

### Research area and journal analysis

**Figure 6:**
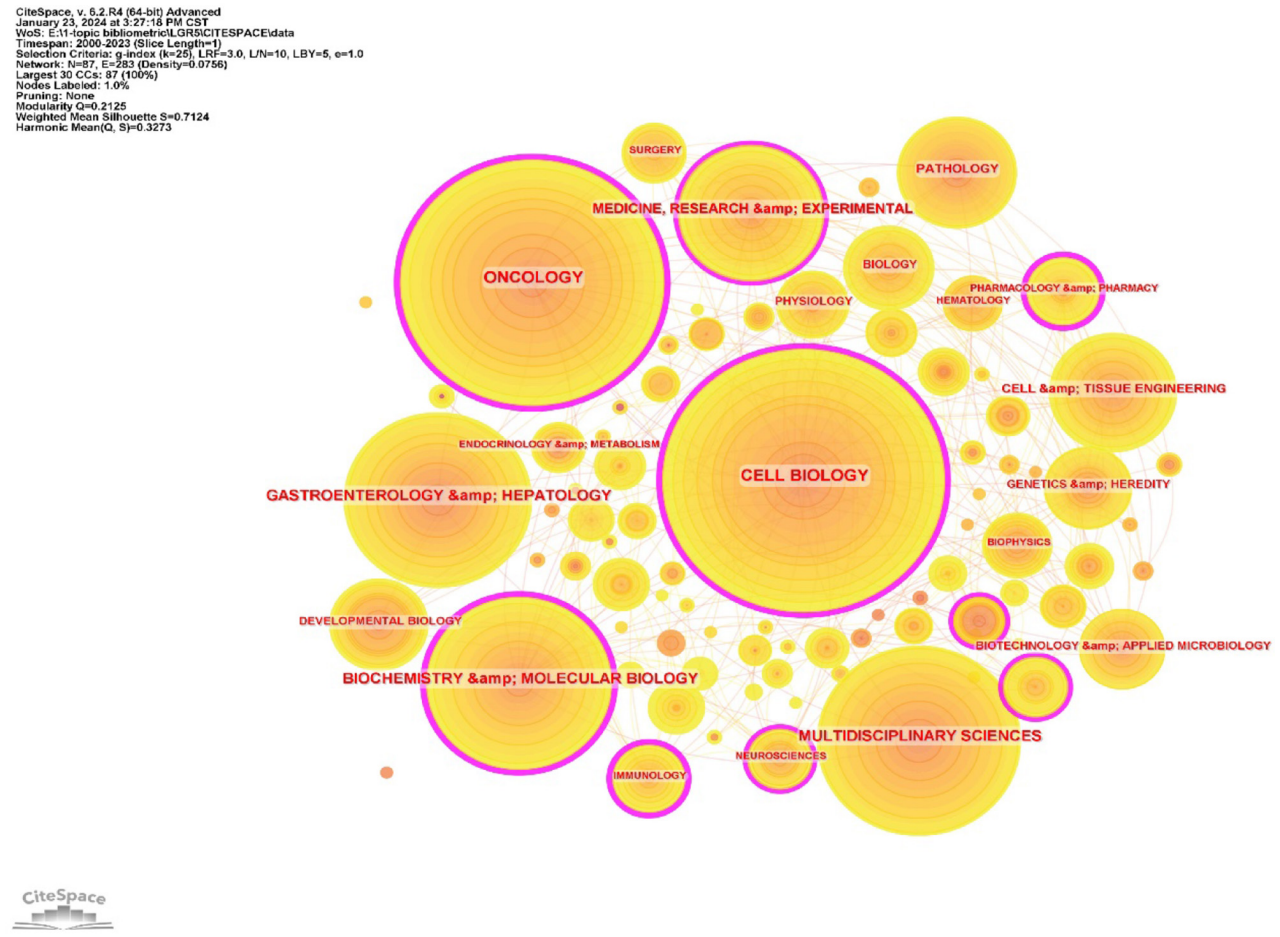
The co-occurrence map of research area. The size of the node represnts the number of articles, and links between countries indicate co-occurrence corrlations, Purple circiles represent high centrality value (>0.1).

The research area analysis indicated that the discipline categories of LGR5 research mainly focused on *CELL BIOLOGY* (525 publications), *ONCOLOGY* (455 publications), and *MULTIDISCIPLINARY SCIENCES* (299 publications), and the centrality value indicated that LGR5 had significant implications in the fields of *CELL BIOLOGY, ONCOLOGY, BIOCHEMISTRY & MOLECULAR BIOLOGY, MEDICINE, RESEARCH & EXPERIMENTAL*, etc. (Figure 6). The most active and influential journals were identified using VOSviewer. A total of 630 journals published articles on LGR5, of which 103 met the threshold of at least five publications (excluding self-citations).

**Figure 7:**
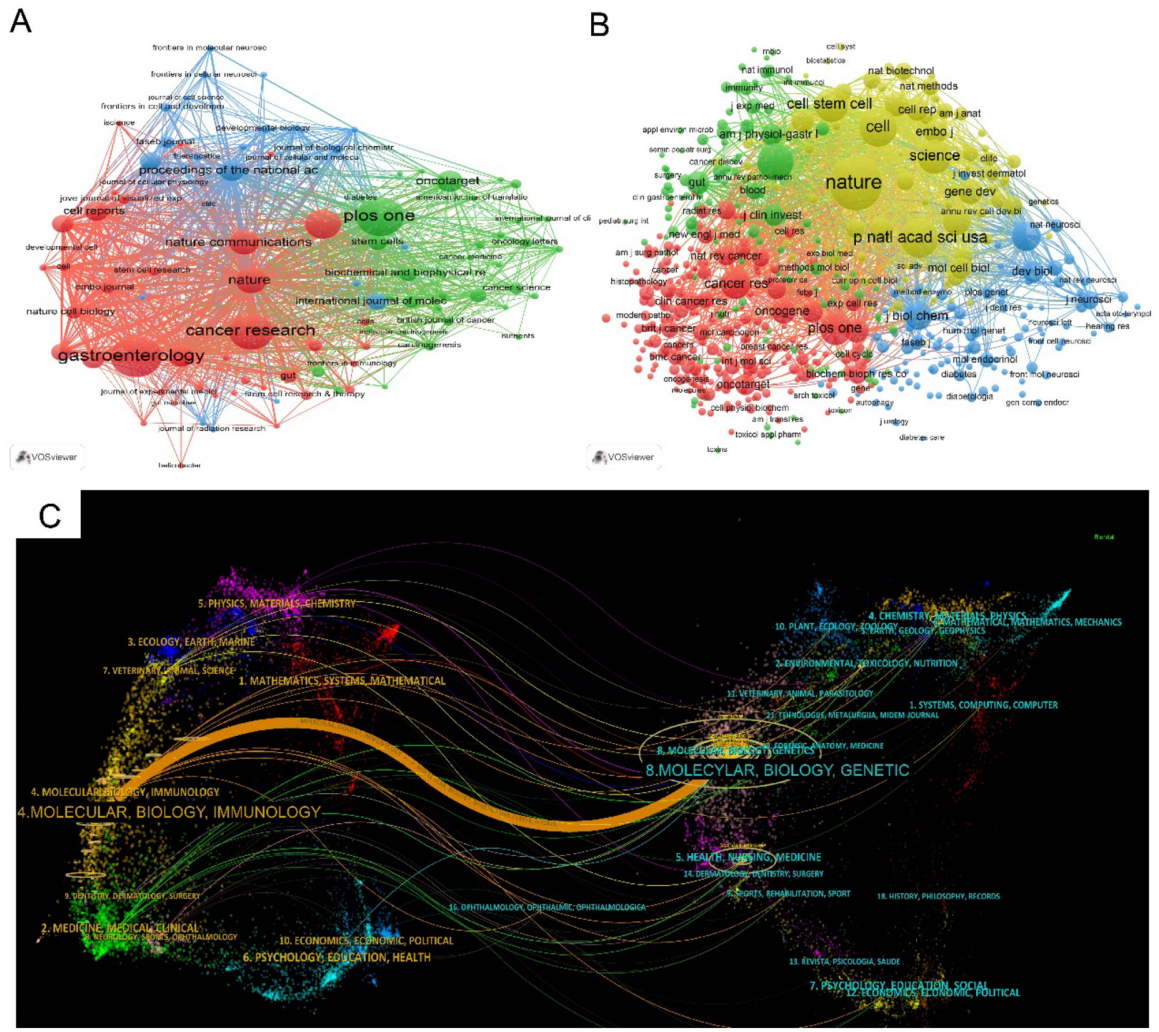
The network visualization mapping of journals **(A)** and co-cited journals **(B)**; Node size reflects frequency, colors denote thematic clusters, and line thickness indicates co-occurrence strength. The dual-map overlay of journals related to LGR5 research **(C)**. The citing journals were at left, the cited journals were on the right, and the colored path represents citation relationship.

The network visualization mapping of journals is shown in Figure 7A; *Gastroenterology* published the most papers (86, 3.4%), followed by *PLoS ONE* and *Cancer Research*. *Nature* revived the most citations (26588) and the greatest total link strength (3,054). Among the top ten journals, seven belonged to the Q1 JCR category, and six hah impact factors greater than 10 (Table 3). Regarding co-cited journals, 4,812 were identified, of which 596 met the threshold of more than 20 citations. The network visualization of co-cited journals is shown in Figure 7B. *Nature* was the most co-citations (7,352 citations), followed by Proceedings of the National Academy of Sciences of the United States of America*, Cell and Gastroenterology*. All 596 co-cited journals had more than 1,000 citations, and among the top ten, nine belonged to Q1 JCR, with eight having impact factors greater than 10 (Table 4).

**Table 3:** The top 10 citation journals ranked by citations.

**No**.	**Journal**	**2023 JCR**	**IF**	**Documents**	**Citations**	**Total link strength**
1	Gastroenterology	Q1	29.4	86	3,506	647
2	Plos one	Q2	3.7	80	2,860	625
3	Cancer research	Q1	11.2	71	1,020	254
4	Scientific reports	Q2	4.6	56	1,439	441
5	Nature	Q1	64.8	47	26,588	3,054
6	Nature communications	Q1	16.6	44	2,640	573
7	Cell stem cell	Q1	23.9	36	6,126	1,097
8	Oncotarget	N/A	N/A	36	971	225
9	*Proceedings of the National Academy of Sciences of the United States of* America	Q1	11.1	36	4,782	877
10	Cell reports	Q1	8.8	33	2,006	499

**Table 4:** The top 10 cited journals ranked by citations.

**No**.	**Journal**	**JCR (2023)**	**IF**	**Citations**	**Total link strength**
1	Nature	Q1	64.8	7,352	311,184
2	*Proceedings of the National Academy of Sciences of the United States of* America	Q1	11.1	3,293	155,137
3	Cell	Q1	64.5	3,159	151,721
4	Gastroenterology	Q1	29.4	2,711	121,051
5	Science	Q1	56.9	2,320	110,918
6	Cell stem cell	Q1	23.9	2,298	108,939
7	Plos one	Q2	3.7	1,917	84,314
8	Cancer research	Q1	11.2	1,657	75,843
9	Development	Q1	4.6	1,512	75,075
10	Nature genetics	Q1	30.8	1,434	63,717

The double overlay map of journals effectively illustrated the citation relationships between citing and cited journals (Figure 7C). Journals were displayed on the left (citing) and right (cited), with citation paths represented by colored lines. One dominant orange citation path was identified, indicating that articles published in Molecular/Biology/Immunology journals were primarily cited by those in *Molecular/Biology/Genetics* journals.

### Pivotal author analysis

**Figure 8:**
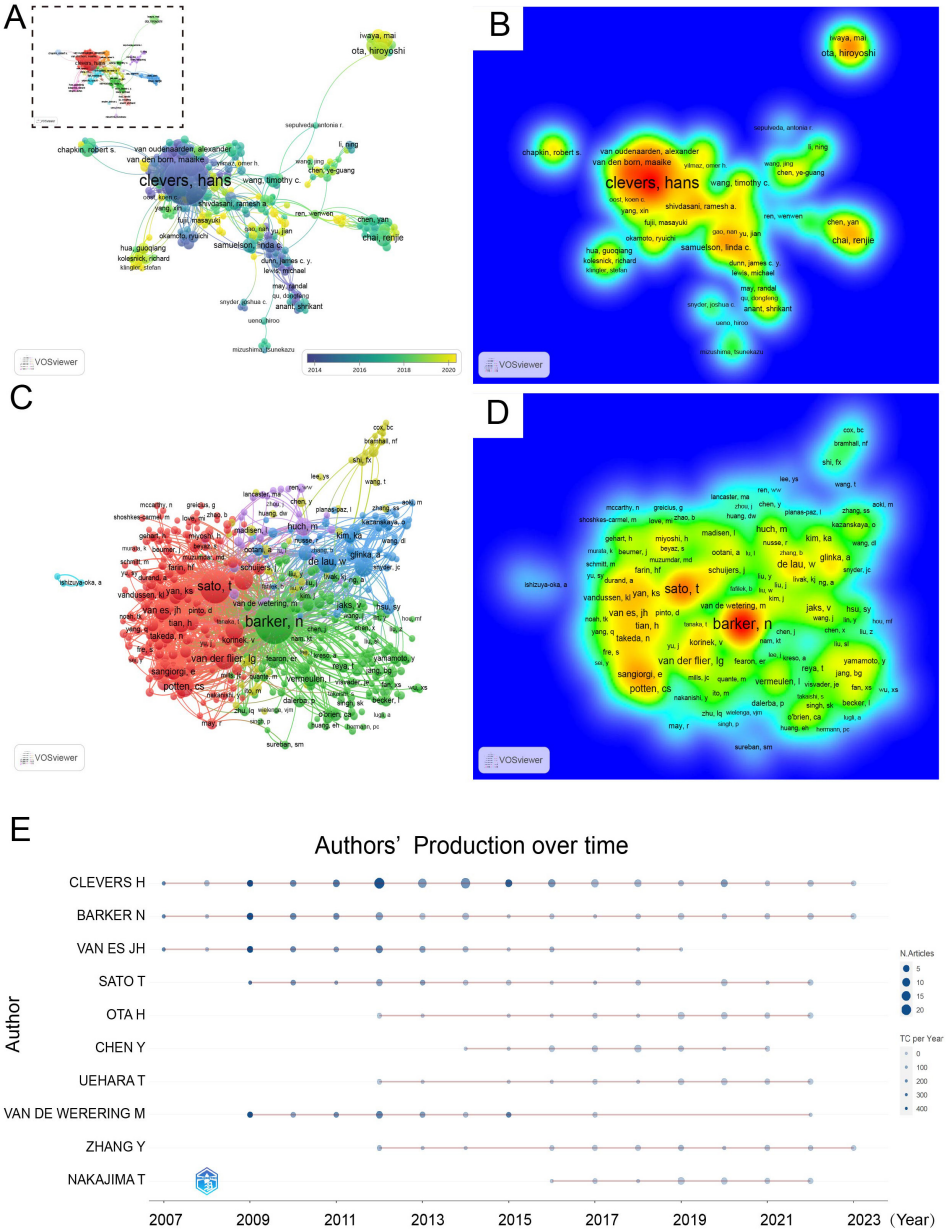
The network visualization **(A)** and density map **(B)** of authors; the network visualization **(C)** and density map **(D)** of co-cited authors; The map of author's production over time **(E)**.

A total of 14,511 co-authorship authors were involved in LGR5 research. After excluding isolated authors, 179 remained connected in the co-authorship network. Visualization analysis revealed 14 clusters, with closely associated groups such as Clevers, H; Barker, N; Van Es, JH; and Ota, H forming the most significant clusters (Figure 8A). Yellow nodes indicated the entry of new researchers. The density map highlighted the most active and influential authors, with color gradients from green to red representing increasing contributions. Clevers, H appeared in the red zone, reflecting his central role (Figure 8B). Of the 42,335 co-cited authors, only two had more than 1,000 co-citations: Barker, N (2,449) and Sato, T (1,406). Authors with at least 605 co-citations (T > 20) were included in the co-cited author network (Figure 8C). Five major clusters were identified: the red cluster led by Sato, T; the green cluster dominated by Barker, N; the blue cluster by De Lau, W; the purple cluster by Huch; and the smallest cluster, composed mainly of Shi, FX and Bramhall, NF. In the density heatmap, red/yellow areas denoted high author concentration and strong collaboration, whereas green/blue areas indicated weaker influence. The density analysis confirmed Barker, N as the most cited author, followed by Sato. In the heatmap, Red/yellow areas mark regions with high author concentration and strong collaboration/impact, while green/blue areas indicate weaker activity. The density map revealed that Barker, N was the most cited author, followed by Sato (Figure 8D). The top ten co-cited authors are listed in Table 5.

**Table 5:** Pivotal authors of documents and top 10 cited authors on LGR5 research.

**No**.	**Authors**	**H_index**	**G_index**	**M_index**	**Citation**	**Documents**	**Cited author**	**Citation**
1	Clevers, H	69	120	3.833	34,416	120	Barker, N	2,449
2	Barker, N	33	54	1.833	20,420	54	Sato, T	1,406
3	Van es, JH	29	30	1.611	22,801	30	Van der Flier, LG	516
4	Sato, T	21	28	1.313	12,600	28	Potten, CS	513
5	Ota, H	5	9	0.385	94	25	De lau, W	496
6	Van, DE Wetering, M	22	23	1.375	18,330	23	Clevers, H	468
7	Chen, Y	15	23	1.364	596	23	Carmon, KS	419
8	Zang, Y	13	23	1	1,420	23	Van es, JH	413
9	Ueharat, T	5	9	0.385	94	23	Snippert, HJ	411
10	Wang, J	14	22	0.875	843	22	Huch, M	357

Clevers, H published the largest number of articles and achieved the highest H-, G-, and M-indices, followed by Barker, N and Van Es, JH, underscoring Clevers' pivotal contributions to the field. The temporal distribution of author productivity is shown in Figure 8E. The timeline bubble chart illustrates each author's research output and impact over time, where bubble size reflects the number of articles and color intensity shows yearly citations (darker means more citations). Notably, Clevers, H and Barker, N maintained continuous productivity from 2007 to 2023, with particularly high output between 2012 and 2014.

### Reference analysis

**Figure 9:**
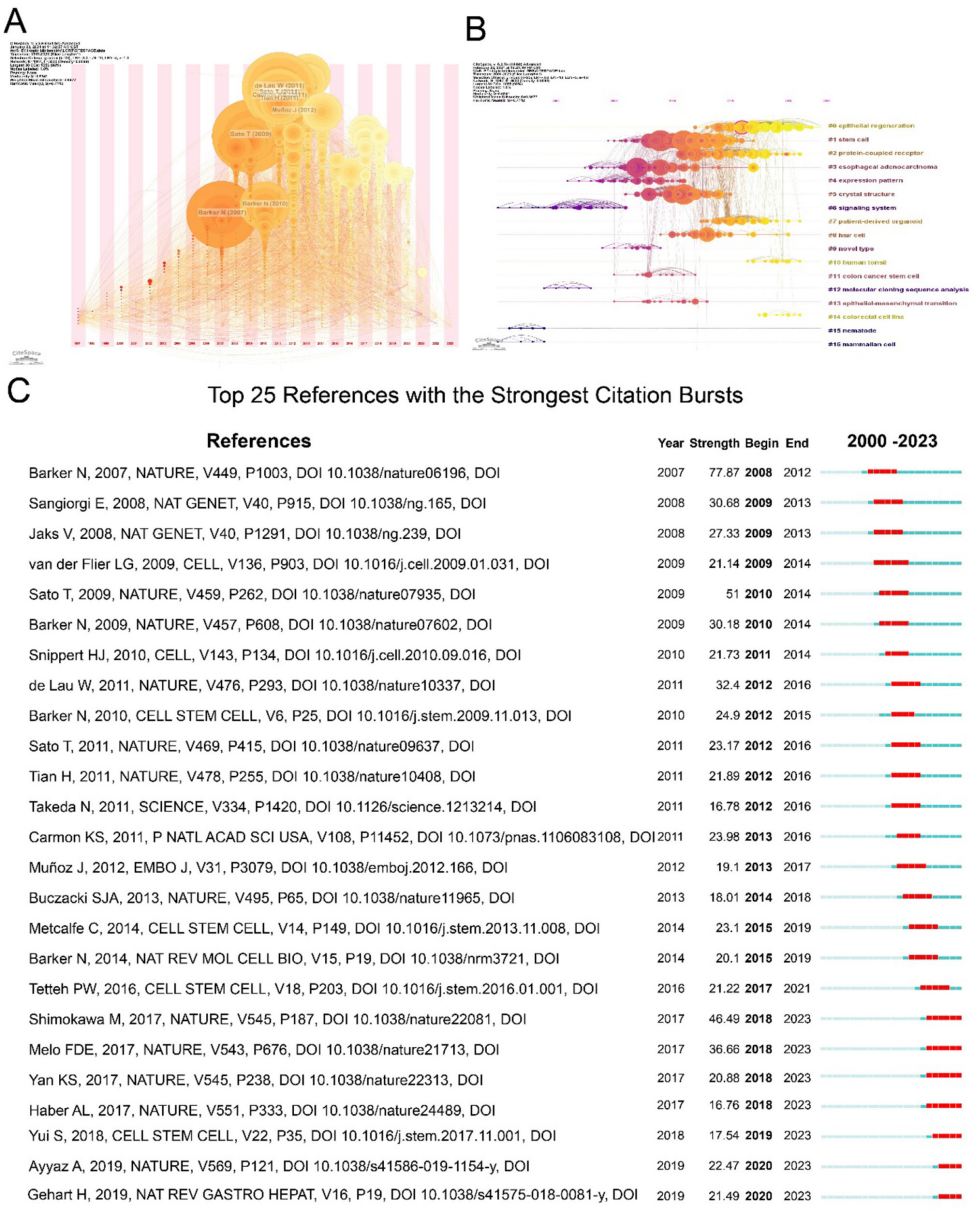
Reference analysis. **(A)** Timeline plot the co-cited reference. **(B)** Timezone plot of the co-cited reference. Each node represents published literature. The vertical axis shows the cluster to which the node belongs, and the horizontal axis of the coordinates shows the time the node was published. The Line between nodes indicates the connection between literatures. **(C)** Top 15 references with the strongest citation bursts.

The top ten most-cited documents are listed in Table 6, providing authoritative references within the field and helping to trace the evolution of past concepts. Notably, nine of these were published by Clevers, including five in *Nature*, three in *Cell*, one in *Annual review of physiology*, and one in *Cell Stem Cell*, with a primary focus on stem cell research. Co-cited references were identified using CiteSpace, and the top ten are listed in Table 7. All were published in *Nature* or *Cell* and had more than 4,000 total citations. Among them, Clevers contributed three papers with an average citation count of 4,997. Among them, Clevers contributed three papers with an average citation count of 4,997. A surge in citations occurred between 2007 and 2011, with representative landmark studies including Barker et al. (2007), Sato et al. (2009), Barker et al. (2010), and de Lau et al. (2011) (Figure 9A). The reference co-citation timeline reveals the temporal evolution of major research themes, with bubbles shifting from purple to yellow to indicate earlier to more recent citations. Clusters #6 and #16 emerged relatively early, whereas clusters #10 and #14 appeared later. Clusters #0, #1, #3, and #5 primarily centered on LGR5. Earlier studies focused on the expression of LGR5 in mammalian cells and its role in signaling pathways, while more recent research hotspots include epithelial regeneration, G protein–coupled receptors, and patient-derived organoids (Figure 9B).

**Table 6:** Top 10 reference with highest citations on LGR5 research.

**No**.	**Title**	**Average citations**	**Corresponding author**	**Journal**	**Total citation**
1	Single Lgr5 stem cells build crypt-villus structures *in vitro* without a mesenchymal niche	277.81	Clevers, H	Nature	4,445
2	Identification of stem cells in small intestine and colon by marker gene *Lgr5*	223.78	Clevers, H	Nature	4,028
3	Paneth cells constitute the niche for Lgr5 stem cells in intestinal crypts	126.71	Clevers, H	Nature	1,774
4	Modeling Development and Disease with Organoids	181.78	Clevers, H	Cell	1,636
5	Crypt stem cells as the cells-of-origin of intestinal cancer	100.88	Clevers, H	Nature	1,614
6	Meta-analysis of genome-wide association data and large-scale replication identifies additional susceptibility loci for type 2 diabetes	85.29	Altshuler, D	Nature genetics	1,450
7	Prospective Derivation of a Living Organoid Biobank of Colorectal Cancer Patients	144.8	Clevers, H	Cell	1,448
8	Intestinal Crypt Homeostasis Results from Neutral Competition between Symmetrically Dividing Lgr5 Stem Cells	91.4	Clevers, H	Cell	1,371
9	Stem Cells, Self-Renewal, and Differentiation in the Intestinal Epithelium	76.38	Clevers, H	Annual review of physiology	1,222
10	Lgr5^+ve^ Stem Cells Drive Self-Renewal in the Stomach and Build Long-Lived Gastric Units *In Vitro*	74.6	Clevers, H	Cell stem cell	1,119

**Table 7:** Top 10 highly co-citation of cited references on LRG5 research.

**No**.	**Title**	**Average citations**	**Corresponding author**	**Journal**	**Total citation**
1	The Hallmarks of Aging	724.41	Kroemer, G	Cell	8,693
2	Signatures of mutational processes in human cancer	543.33	Clevers, H	Nature	6,520
3	Comprehensive Integration of Single-Cell Data	1,006	Satija, R	Cell	6,036
4	Finding the missing heritability of complex diseases	337.41	Visscher, PM	Nature	5,736
5	Comprehensive molecular characterization of human colon and rectal cancer	375.46	Thomson, E	Nature	4,881
6	mTOR Signaling in Growth, Metabolism, and Disease	599.87	Sabatini, DM	Cell	4,799
7	Single Lgr5 stem cells build crypt-villus structures *in vitro* without a mesenchymal niche	277.81	Clevers, H	Nature	4,445
8	Wnt/β-Catenin Signaling and Disease	320.46	Nusse, R	Cell	4,166
9	Wound repair and regeneration	242.48	Longaker, MT	Nature	4,122
10	Identification of stem cells in small intestine and colon by marker gene Lgr5	223.78	Clevers, H	Nature	4,028

References with citation bursts are defined as those cited intensively within a specific time period. By setting the burst duration to at least 2 years, we identified 25 references with the strongest citation bursts (Figure 9C). The paper titled “*Identification of stem cells in small intestine and colon by marker gene LGR5”* (Xu et al., 2022), published in *Nature* by Barker N in 2007, exhibited the strongest citation burst between 2008 and 2012. Notably, seven references (28%) continued to show citation bursts until 2023, indicating sustained scholarly interest in LGR5 research.

### Keywords analysis

**Figure 10:**
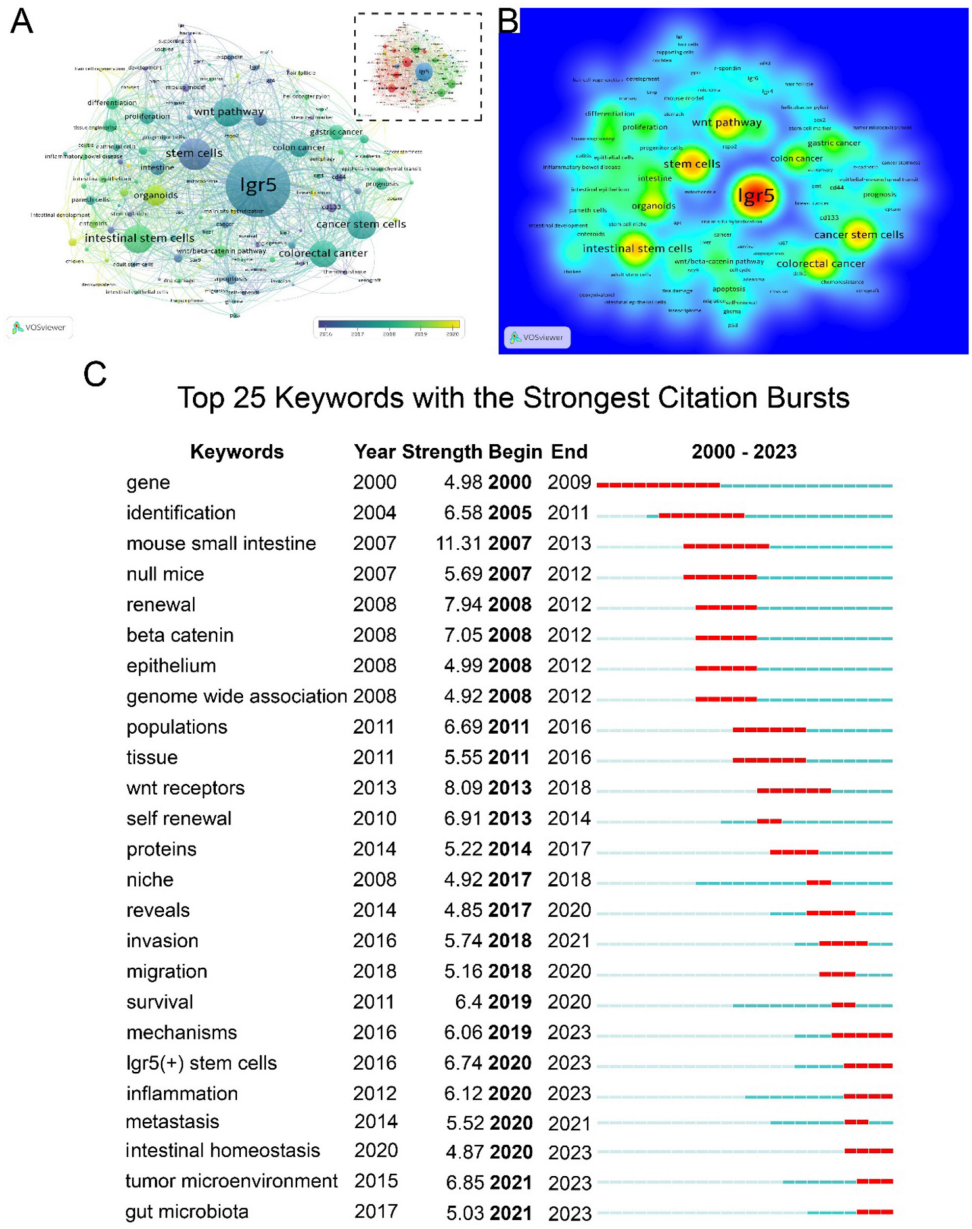
Keyword co-occurrence, clustering, and development. **(A)** The network and overlay visualization of LGR5 research; **(B)** Density map of LGR5 research; **(C)** Top 25 keywords with the strongest citation bursts.

In this study, 3,468 keywords were identified, of which 165 appeared more than five times. Keyword network, overlay, and density maps were used to highlights research hotspots and emerging keywords in specific academic fields. In Figure 10A the color gradient from purple to yellow corresponds to the time interval of 2016 to 2020. Cluster analysis provided a conceptual framework comprising four cluster: cluster 1 (red, 46 items) focused primarily on LGR5 expression; cluster 2 (green) on the functional roles of LGR5; cluster 3 (blue) on hair follicles and mouse models; and cluster 4 (yellow) on mechanistic studies. Core terms included *LGR5, stem cells, Wnt pathway, cancer stem cells, gastric cancer*, and *intestinal stem cells* (Figure 10B). As shown in Table 8, keywords were divided into four clusters according to their attributes: key molecules, state/pathway, disease, and cell type, which may indicate mainstream themes and frontiers in a specific research field. The top three molecules were LGR5, β-catenin, and CD133. The most frequent pathways were the *Wnt pathway, proliferation*, and *regeneration*. Commonly studied diseases included colorectal cancer, gastric cancer, and inflammatory bowel disease. The most relevant cell types were *intestinal stem cells, organoids*, and *Paneth cells*. As shown in Figure 10C, keywords with the strongest citation bursts revealed “that the mouse small intestine” had the longest burst length (11.31), spanning 2007–2013. Other burst terms such as *renewal, Wnt receptor, self-renewal*, and *LGR5 (*+*) stem cells* reflected early emphasis on stemness characteristics. More recently, keywords such as *mechanisms of LGR5 (*+*) stem cells, inflammation, intestinal homeostasis, tumor microenvironment*, and *gut microbiota* have attracted increasing attention, suggesting they may evolve into dominant research trends in LGR5 studies.

**Table 8:** Top 10 key molecules, states/pathway, diseases, and cell types in studies on LGR5.

**No**.	**Molecule**	**Occurrence**	**State/Pathway**	**Occurrence**	**Disease**	**Occurrence**	**Cell type**	**Occurrence**
1	LGR5	379	Wnt pathway	193	Colorectal cancer	185	Intestinal stem cells	153
2	β-catenin	78	Proliferation	52	Gastric cancer	95	Organoids	132
3	CD133	26	Regeneration	43	Inflammatory bowel disease	77	Paneth cells	94
4	CD44	22	Differentiation	42	Hepatocellular carcinoma	66	Epithelial cells	86
5	R-Spondin	17	Prognosis	37	Necrotizing enterocolitis	64	Enteroids	85
6	BMI1	16	Apoptosis	32	Breast cancer	58	Progenitor cells	83
7	LGR6	15	Notch signaling	31	Ovarian cancer	54	Adult stem cells	79
8	LGR4	13	Inflammation	29	Glioma	35	Intestinal epithelial cells	74
9	p53	13	Tumorigenesis	29	Intestinal injury	32	Goblet cells	64
10	DCLK1	8	EMT	28	Type 2 diabetes	13	Hair cells	45

## Discussion

### General information

Over the past decade, the LGR5-related publications have steadily increased, highlighting growing scholarly attention and continuous improvement in this research field. Annual publications are a key indicator of research dynamics. LGR5 research involved three 3 stages: germination stage (2000 to 2010), focusing on the structure, function and expression; the rapid growth stage (2011 to 2019), when 1,362 articles were published, which was approximately fifteen times of the number of articles published in the germination stage; and platform stage (2020 to 2023), characterized by stable but sustained high activity.

Research on LGR5 has been conducted worldwide, reflecting broad global interest. The USA, China, and Japan were the most prolific contributors. The USA exhibited the highest centrality value, single-country publications (SCP), and multiple-country publications (MCP), underscoring its leadership. The Netherlands, the earliest country to conduct this research and had the largest burst length, laying a solid foundation for subsequent research. Notably, since 2022, China has surpassed the United States in annual publications, driven by its large researcher base and growing investment. I notably, although U.S. institutions such as Harvard University, the University of California System, and the University of Texas System demonstrated high centrality, the most cited institutions were located in the Netherlands. Overall, the United States is dominant, while the Netherlands has been the most influential. Several articles were mainly published in journals related to cell biology and oncology, indicating that LGR5 plays a vital role in biological processes and cancer progression. Journal and co-cited journal analyses showed that *Gastroenterology* published the largest number of articles, whereas *Nature* received the largest number of co-cited references. *As a leading journal in digestive disease research, Gastroenterology emphasizes original studies on gastrointestinal pathophysiology and treatment, reinforcing the importance of LGR5 in gastrointestinal tissues*. The dual-map overlay of journals represents the distribution of topics in academic journals (Xu et al., 2022). There was only one citation path from Molecular/Biology/Immunology journals to Molecular/Biology/Genetic journals, indicating a sustained focus on basic research. In addition, journals in JCR Q1 category accounted for the majority of the top ten journals (70%) and co-cited journals (90%), implying that these journals are of interest and play a major role in LGR5 related research.

Highlighting the contributions of influential researchers, for example, the authors who published the most articles or were most co-cited by other scholars, can provide guidelines and further directions for scholars who are starting or conducting in-depth research and also provide effective resources and trends (Yeung et al., 2017). Prolific or influential researchers mostly belong to organizations with high citations, suggesting frequent cooperation and communication among influential authors (Ou et al., 2023). In our analysis, Clevers H from the Netherlands published the most articles with the highest H, G, and M indices and citations. Since LGR5 was identified in the USA in 1998, its expression pattern and function have been extensively studied, especially by Clevers, H (de Lau et al., 2014; Hsu et al., 1998; Sato et al., 2009; Barker et al., 2007; Shimokawa et al., 2017). The most co-cited author was Barker from the Netherlands, followed by Sato, also from the Netherlands, and then Clevers H from the USA. Clevers authored nine of the ten most-cited references, published in *Nature, Cell, Annual Review of Physiology*, and *Cell Stem Cell*, highlighting his pivotal role. This implies that Clevers, H and his team contributed significantly to LGR5 research. The map of authors and co-cited authors offers valuable information on potential collaborators and research teams.

### Knowledge base

Generally, the knowledge base refers to the body of literature cited by the research community related to similar research fields (Li et al., 2018). Highly co-cited references often reflect research hotspots. In our analysis, the most co-cited references were published between 2007 and 2012. An article titled “Single LGR5 stem cells build crypt-villus structures *in vitro* without a mesenchymal niche” published in 2009 in *Nature*, received the highest citation frequency of 4,445 citations and demonstrated that LGR5 (+) stem cells can generate crypt-villus organoids and maintain their structure hierarchy (Sato et al., 2009). The second co-cited article was published by Clevers, H in *Nature* in 2007, titled “Identification of stem cells in the small intestine and colon by marker gene *LGR5*.” This study identified *LGR5* as a molecular marker for stem cells in multiple adult tissues and cancers (Barker et al., 2007). Another influential study, “Paneth cells constitute the niche for LGR5 stem cells in intestinal crypts,” published in Nature in 2011, revealed the essential role of Paneth cells in sustaining LGR5(+) stem cells. The fourth co-cited review was published in *Cell* by Clevers, H, titled “Modeling Development and Disease with Organoids.” and summarizes the potential applications of organoid cultures in models of human organ development and various human pathologies (Clevers, 2016). In the article titled “Crypt stem cells as the cells-of-origin of intestinal cancer,” which was the fifth co-cited paper, Clevers, H investigated the role of APC in the development of intestinal cancer using an LGR5 knock-in mouse model (Barker et al., 2009). The sixth co-cited paper was published by Altshuler, D in *Nature genetics*, titled “Meta-analysis of genome-wide association data and large-scale replication identifies additional susceptibility loci for type 2 diabetes.” This study identified at least six loci and estimated their value in understanding the inherited basis of type 2 diabetes (Zeggini et al., 2008). Clevers, H published the seventh co-cited article titled “Prospective derivation of a living organoid biobank of colorectal cancer patients,” reporting the establishment of tumor organoid culture from 20 CRC patients. This study demonstrates the potential of organoid technology in cancer genetics and patient trials (van de Wetering et al., 2015). In 2010, Clevers, H published the eighth paper titled “Intestinal crypt homeostasis results from neutral competition between symmetrically dividing LGR5 stem cells” in *Cell*. This study showed that LGR5 stem cells undergo symmetrical division through neutral competition to stabilize the intestinal crypt and described their patterns of division (Snippert et al., 2010). The ninth paper was published by Clevers, H in *Annual review of physiology*. This review summarized intestinal stem cell markers, the identification of intestinal stem cells, genetic practices in intestinal development, and the aberrant deregulation of these molecular pathways that result in colon cancer (van der Flier and Clevers, 2009). The tenth co-cited reference titled “LGR5(+ve) stem cells drive self-renewal in the stomach and build long-lived gastric units *in vitro*”, was published in *Cell stem cell* by Clevers, H. This study demonstrated the application of LGR5 (+) stem cells in gastric epithelial renewal, inflammation, and cancer (Barker et al., 2010). The top ten co-cited reference were mainly focused on LGR5 stem cells, crypt-villus, Paneth cells, organoids, and self-renewal, which represent the expression pattern and function of LGR5.

Overall, the top ten co-cited references converged on themes of LGR5(+) stem cells, crypt-villus structures, Paneth cells, organoids, and self-renewal. These studies, largely published between 2007 and 2012, laid the foundation for understanding LGR5 expression patterns, intestinal development, and its roles in gastrointestinal diseases, particularly cancer, consistent with the findings of the time-zone map.

### Hotspot and emerging trend

In bibliometric analysis, keyword co-occurrence and citation bursts reveal research, while time-zone plot illustrate their evolution (Zhang et al., 2021). Our analysis identified stem cells, cancer stem cells, intestinal stem cells, Wnt pathway, colorectal cancer, and organoids as the most frequent terms highlighting their centrality in LGR5 research. In the germination stage (2000–2010), burst terms included gene, identification, mouse small intestine, renewal, and β-catenin. Significantly, the mouse small intestine may have been the point with the strongest citation bursts. In the rapid growth stage (2011–2019), the rising terms included mechanism investigation, including Wnt receptor, mechanism, population, invasion, migration, niche, protein, TME, and LGR5 (+). Intestinal homeostasis emerged as a key topic in the platform stage since LGR5 (+) stem cells play a vital role in intestinal development, homeostasis, and injury-mediated regeneration. The proper function of LGR5 (+) stem cells depends on niche factors, such as lymphatic endothelial cells and rspo3+GREM1+fibroblasts (Goto et al., 2022).

The TME had the highest citation bursts in recent years according to keyword citation burst analysis. Sustained interactions between tumor cells and the TME play a decisive role in the occurrence, development, metastasis, and response to tumor treatment. The TME has attracted widespread research and clinical interest (Xiao and Yu, 2021). Recently, a study found that LGR5-marked cells represent an important tumor-initiating cell population, and cancer-associated fibroblasts (CAFs) showed robust effects on tumor-initiating cells marked by LGR5. This demonstrates that the effect of the TME on stem cell-related therapies suggests the possibility of combining CAF-targeted therapy with tumor stem cell-targeted therapy for liver cancer treatment (Zhang et al., 2023). In inflammatory bowel disease, the dysregulation of LGR5 (+) stem cells leads to the disruption of intestinal homeostasis with a deficiency in epithelial crypt structures and tissue regeneration (Kwon et al., 2024). An article titled “Visualization and targeting of LGR5^+^ human colon cancer stem cells” (Shimokawa et al., 2017), which revealed that LGR5 (+) cells serve as cancer stem-like cells (CSCs) in CRC and play an important role in cancer regrowth, had the highest citation bursts in recent years, followed by an article titled “A distinct role for LGR5^+^ stem cells in primary and metastatic colon cancer” (de Sousa e Melo et al., 2017), which demonstrated that depletion of LGR5 (+) stem cells restricts primary cancer growth. However, LGR5 (+) stem cells are replenished with LGR5 (+) cells, leading to cancer metastasis. Targeting CSCs may be a potential therapeutic strategy for the treatment of metastatic cancer. The initial characterization of LGR5 as an intestinal stem cell marker has been studied for 10 years, and LGR5 targeted therapies have not yet reached the clinical stage for CRC owing to contradictory results. Similar to normal gut homeostasis, LGR5 is functionally redundant at certain stages of tumor growth, but at specific phases of tumor growth, especially during metastatic progression and drug/radiation exposure, LGR5 becomes essential and LGR5-CRC cells rapidly acquire LGR5 expression. Thus, it is important to conduct tumor stage-specific assessments of LGR5 therapies in *in vivo* models of metastases (Morgan et al., 2018; Kobayashi et al., 2012).

LGR5, as a well-recognized stem cell marker, exhibits diverse expression patterns and functional roles across different cancer types. In colorectal cancer, LGR5 is often highly expressed in tumor-initiating cells and is associated with enhanced tumorigenicity, chemoresistance, and poor prognosis (Morgan et al., 2018). In gastric cancer, LGR5 expression shows spatial heterogeneity, and its high expression has been linked to increased proliferation and invasion, although some studies report context-dependent effects on patient outcomes (Simon et al., 2012; Ehara et al., 2021). In hepatocellular carcinoma (HCC), LGR5 marks a distinct tumor cell subpopulation with stem-like properties, contributing to tumor progression and metastasis, and has been proposed as a potential biomarker for precise staging (Cao et al., 2020). These differences highlight that the functional significance and clinical implications of LGR5 are highly tumor-specific, emphasizing the need to interpret its role within the context of each cancer type.

**Figure 11:**
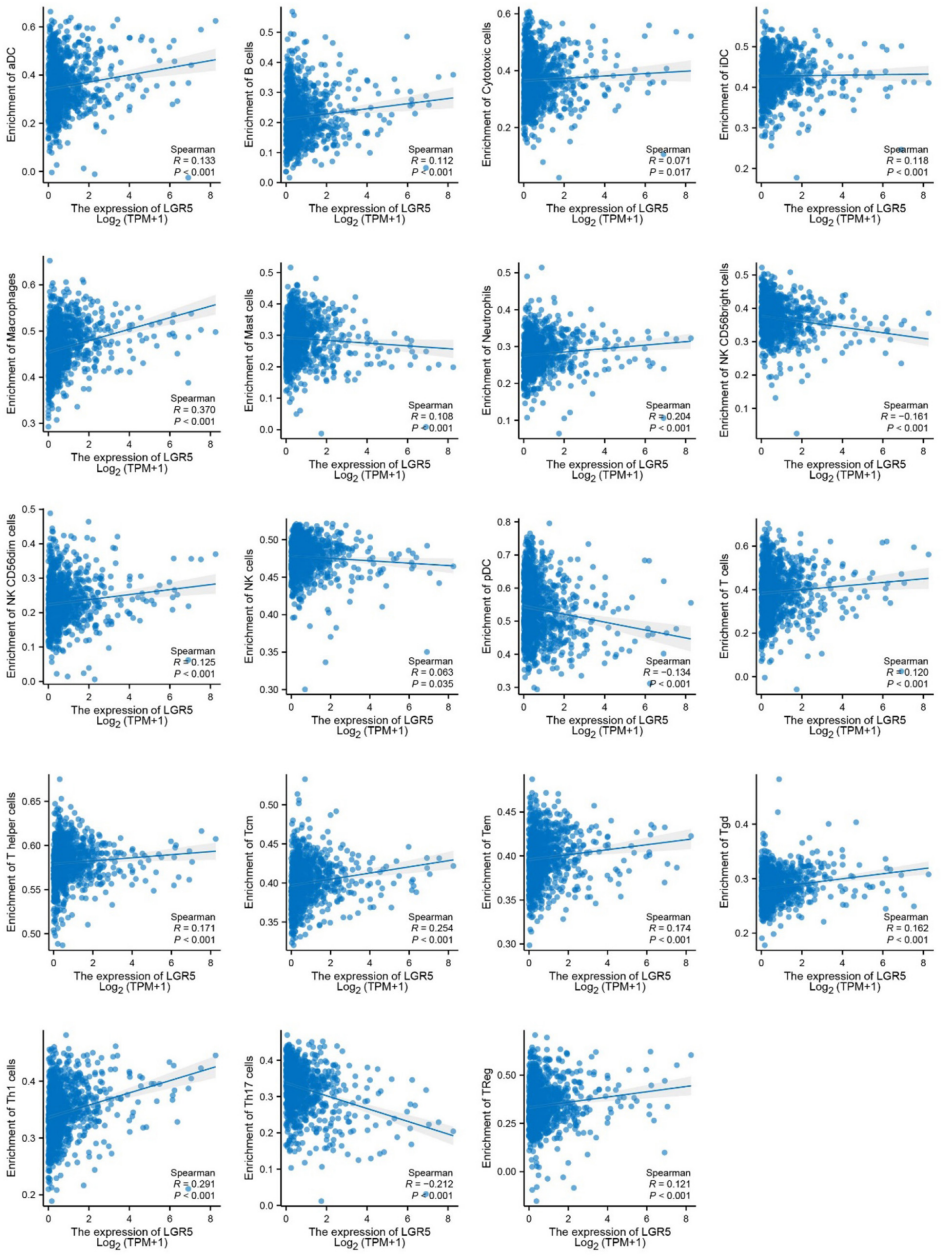
Correlation between LGR5 expression and immune cell infiltration in tumors. Scatter plots showing the correlations between *LGR5* expression (log2 TPM+1) and the enrichment of multiple immune cell types, including dendritic cells (DC), B cells, cytotoxic cells, CD8^+^ T cells, macrophages, mast cells, neutrophils, NK cells, pDC, T helper cells, Tcm, Tgd, Th1, Th17, and Treg cells. Correlations were assessed using Spearman's correlation analysis, with correlation coefficients (R) and *P*-values shown in each panel.

In this study, we used CIBERSORT to analyze the correlation between LGR5 expression and immune cell infiltration in the tumor microenvironment. The results showed that LGR5 expression was significantly positively correlated with the infiltration of macrophages and T cells, particularly central memory T cells (Tcm), T helper cells, and regulatory T cells (Treg), suggesting that tumors with high LGR5 expression may be more likely to attract or sustain these immune cell populations. Conversely, LGR5 expression showed a mild negative correlation with NK CD56 bright cells, plasmacytoid dendritic cells (pDCs), and Th17 cells, indicating that these immune populations may be relatively reduced in tumors with high LGR5 expression. Overall, LGR5 appears to exert a complex regulatory effect on immune infiltration, displaying both positive and negative associations, which suggests that it may modulate the immune composition of the tumor microenvironment rather than acting in a unidirectional manner (Figure 11).

Overall, we found that LGR5 research initially focused on the expression pattern and function of LGR5 digestive system; subsequently LGR5 mechanism in intestinal development and digestive system tumor initiation became the hot topics. Furthermore, targeting LGR5 (+) stem cells in a specific phase of cancer or exploring effective strategies for targeting CSCs, such as TME combination targeting, could be the hotspots in the near future.

### Highlight the role of LGR5 in TME

In terms of the highest citation burst value in keyword citation burst analysis, the TME attracted significant attention. The TME is an integral part of cancer, and the discovery of novel targets within the TME can help guide and improve the efficacy of various cancer therapies (Pitt et al., 2016). Studies have shown that CAFs transformed by cancer cells within the TME secrete interleukin-6 and enhance STAT3 signaling in colon cancer cells, promoting the production of CSCs and further driving the progression of malignant tumors (Huang et al., 2019). For example, co-culture with CAFs increases the expression of LGR5 and β-catenin and reduces 5-FU resistance in CRC (Yadav et al., 2020). CAFs also promote LGR5-marked liver tumor-initiating cells and influence CSC-targeted therapy (Zhang et al., 2023). Additionally, αSMA+ fibroblasts reduce LGR5 (+) cancer stem cells and inhibit CRC progression (McAndrews et al., 2021). CAST is a potential cancer promoter in macrophage-infiltrated gastric cancer and regulates LGR5 expression.

These studies have revealed that the composition of the TME affects the occurrence and development of tumors, drug resistance, and immune responses by influencing LGR5 expression or LGR5 (+) stem cells. Moreover, nutrient stress in the TME can alter the stability of LGR5 protein and its membrane localization via the modulation of LGR5 glycosylation status, which demonstrates that the TME could affect LGR5 function and regulate Wnt signaling (Morgan et al., 2015). The cancer stem cell marker LGR5 is associated with immune-related TME. LGR5 expression is upregulated in gastric cancer (GC) cells co-cultured with regulatory T cells (Tregs) or treated with exogenous TGF-β1. TGF-β1 increased LGR5 and β-catenin expression via the TGF-β1 signaling pathway, which proves that LGR5 plays an important role in cancer immunosuppressive microenvironment mediated by Tregs and TGF-β1. However, further research is needed to determine the exact molecular mechanism of LGR5 and the the TME.

### Limitations

This is the first bibliometric study on LGR5 research, but it had some limitations. First, the data analyzed in this study were retrieved exclusively from the Web of Science Core Collection. While WoSCC is one of the most academically recognized and influential databases for bibliometric analysis, offering reliable quantitative indicators such as publication counts and citation frequencies, it may not include all relevant publications indexed in other sources such as Scopus or PubMed. Future studies could integrate multiple databases to provide a more comprehensive overview of the field. Second, this study mainly focused on original articles and reviews; other types of publications, such as editorial materials and letters, were excluded. However, original articles and reviews are the main types of publications and can provide more mechanistic research content for researchers (Xu et al., 2022).

Another limitation of this study is that the dataset was restricted to publications from 2000 to 2023. This was due to the time lag between data collection and the submission/review process. As a result, more recent publications from 2024 were not included. We acknowledge this limitation and note that future bibliometric updates may incorporate publications from 2024 and beyond, which would provide a more comprehensive overview of the evolving research landscape.

## Conclusions

A bibliometric cluster analysis of LGR5 research clarifies the distribution and development trends of related research hotspots. Research on LGR5 continues to develop through active global collaboration. The USA holds a dominant position in this research while the Netherlands was the earliest country to conduct this research. The most influential author, Cleavers, from the Hubrecht Institute (KNAW) of the Netherlands published many papers with high impact factors. LGR5 and its role in stem cells were the primary hotspots. “Inflammation” and “tumor microenvironment” showed the strongest burst in recent years, indicating the potential trend for the future research.

In addition to the bibliometric findings, experimental studies have highlighted the biological and translational relevance of LGR5. As a stem cell marker, LGR5 regulates Wnt/β-catenin signaling and contributes to tumor initiation, progression, and therapy resistance in cancers such as colorectal, gastric, liver, and ovarian cancer (Wang et al., 2025; Liu et al., 2018). Preclinical studies, including antibody-based and CAR-T approaches, have shown promising antitumor activity against LGR5-positive cells, though challenges such as tumor heterogeneity and potential toxicity to normal stem cells remain (Mueller et al., 2025). These findings complement our bibliometric analysis and highlight the need for integrated research to further elucidate the mechanistic roles and therapeutic potential of LGR5.
